# Hippocampal volumes and cognitive performance in children born extremely preterm with and without low-grade intraventricular haemorrhage

**DOI:** 10.1007/s00429-023-02643-w

**Published:** 2023-04-21

**Authors:** L. Fernández de Gamarra-Oca, H. Kvanta, L. Broström, D. Nosko, E. Eklöf, N. Ojeda, L. Zubiaurre-Elorza, N. Padilla, U. Ådén

**Affiliations:** 1 Department of Psychology, Faculty of Health Sciences, University of Deusto, Avda de Las Universidades 24, 48007 Bilbao, Bizkaia Spain; 2 Department of Women’s and Children’s Health, Karolinska Institute, Stockholm, Sweden; 3 Sachs’ Children and Youth Hospital, Södersjukhuset, Stockholm, Sweden; 4 Department of Paediatrics, Örebro University Hospital, Örebro, Sweden; 5 Department of Neonatal Medicine, Karolinska University Hospital, Stockholm, Sweden; 6 Department of Biomedical and Clinical Sciences, Linköping University, Linköping, Sweden

**Keywords:** Intraventricular haemorrhage, Extremely preterm, Cognition, Childhood

## Abstract

Children born extremely preterm, especially those with intraventricular haemorrhage (IVH), are at increased risk of adverse cognitive outcomes during childhood. The present study aimed to explore the effects of IVH (grades I–II) on hippocampal volumes, and their correlates with cognitive performance. The sample consisted of 94 participants, including 54 children born extremely preterm (19 with IVH, grades I–II), and 40 children born at term. All participants underwent a magnetic resonance imaging study at the age of 10 (*M*_age_ = 10.20 years; SD_age_ = 0.78), and 74 of them (45 extremely preterm and 29 full-term) carried out a cognitive assessment at 12 years old. Children born extremely preterm had lower scores in cognitive performance compared to their full-term peers. Significant positive partial correlations were observed between global bilateral hippocampus, left CA-field, and left subiculum volumes with processing speed in the full-term group, while no significant correlations were found in the extremely preterm group. Moderation analyses in the extremely preterm sample revealed that low-grade IVH moderated the relationship between right hippocampal volume and full-IQ (*F*_(4,40) = _5.42, *p* = 0.001, *R*^2^ = 0.35). Having greater right hippocampal volume had a protective effect on full-IQ in those children born extremely preterm with low-grade IVH.

**Supplementary Information:**

The online version contains supplementary material available at 10.1007/s00429-023-02643-w.

Children born extremely preterm (< 28 weeks of gestation) in the twenty-first century are more likely to survive than those born during an earlier era of prenatal and neonatal intensive care. Nevertheless, long-term neurosensory, cognitive, academic achievement, and motor outcomes remain a matter of concern in children born extremely preterm (Cheong et al. 2020). Along with the presence of immaturity at birth, intraventricular haemorrhage (IVH) is a major problem in preterm newborns, since the majority have adverse neurodevelopmental consequences (Garvey et al. 2022). Although low grades of IVH had been related to increased rates of neurosensory impairment, developmental delay, cerebral palsy, and poorer neurodevelopmental outcomes (Bolisetty et al. 2014; Hollebrandse et al. 2021; Klebermass-Schrehof et al. 2012; Patra et al. 2006); grade 3–4 and the presence of white matter injury and/or ventriculomegaly are clearly related to worse prognosis (Vohr 2022). For instance, in rabbit pups, long-term risks after IVH entail wide-ranging alterations in cortical organisation and microstructure (Romantsik et al. 2022). In very preterm infants with low-grade IVH, cortical underdevelopment, functional impairment, and microstructural immaturity have been found at term-equivalent age (Argyropoulou et al. 2020).

A range of perinatal events that affect preterm newborns have been related to smaller hippocampal volume at 2 years of age (Thompson et al. 2008). Decreased hippocampal volume has also been linked to domain-specific neurodevelopmental impairments in preterm infants with perinatal brain damage (Strahle et al. 2019). The hippocampus has a significant impact on memory and learning and is closely linked to the ventricular system, which makes it a possible location of IVH-induced injury (Garton et al. 2016). Volumetric reductions in the hippocampus have been specifically associated with preterm children with germinal-matrix IVH (Fernández de Gamarra-Oca et al. 2021). However, smaller left dentate gyrus volume has been linked to poorer visual working memory in preterm-born children (i.e., 25–34 weeks of gestation) without brain injury (Aanes et al. 2019).

After extremely preterm delivery, considering the increased prevalence of intellectual impairment (O’Reilly et al. 2020) and the altered brain growth in the absence of focal brain lesions at term-equivalent age (Padilla et al. 2015), this MRI study assesses the role that hippocampal volumes may play in the cognitive performance of children born extremely preterm with and without low-grade IVH. Hence, three hypotheses are proposed. First, children born extremely preterm will have reduced global and regional hippocampal volumes in comparison to the full-term group. Second, smaller global and regional hippocampal volumetric values will be related to cognitive performance in children born extremely preterm. And third, the presence of low-grade IVH will moderate the relationship between global hippocampal volumes and cognition in children born extremely preterm.

## Methods

### Participants

**Fig. 1:**
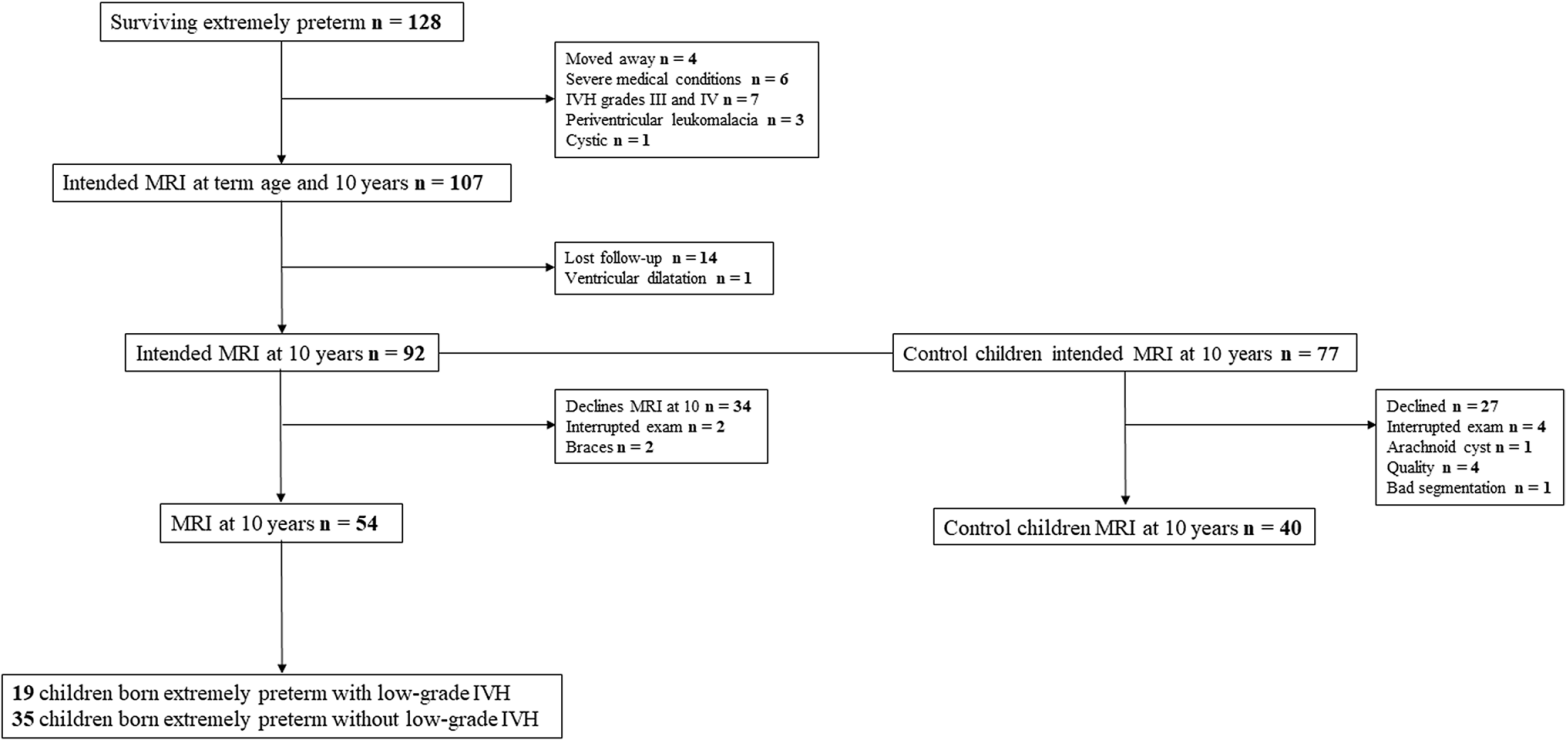
Flow chart for children born extremely preterm with and without intraventricular haemorrhage I-II and full-term controls. Children born extremely preterm and full-term controls who had an MRI scan when they were 10 years old

Extremely preterm birth was defined as birth up to gestational age (GA), 26 weeks, and 6 days (EXPRESS study) (Serenius et al. 2013). All extremely preterm neonates born alive in Stockholm between 1 January 2004 and 31 March 2007 were included in the study (*n* = 191). A total of 128 newborns (67.0%) reached term-equivalent age (GA of 40 weeks and 0 days). The exclusion criteria were lack of parental consent, severe medical conditions, major brain lesions [cystic periventricular leukomalacia, IVH grade III and IV diagnosed with cranial ultrasound during the neonatal period, focal brain lesions, cysts and severe white matter abnormalities on MRI as defined by a previously published scoring system (Inder et al. 2003)], and low-quality MRI images (defined as incomplete coverage of the brain, motion artefacts, or blurring of the grey and white matter interfaces). After exclusions, high-quality MRI data were obtained for 54 children born extremely preterm at age 10 (*M*_age = _10.20 years) (see Table 1). More specifically, 19 children born extremely preterm with low-grade IVH and 35 children born extremely preterm without neonatal brain injury were included in the study (see Fig. 1).

**Table 1:** Clinical data of children born extremely preterm with and without intraventricular haemorrhage I–II

	Children born extremely preterm with IVH I–II *n* = 19	Percentage (%)	Children born extremely preterm without IVH *n* = 35	Percentage (%)	*P* value
BW (g), mean ± SD	0.792 ± 0.149	–	0.861 ± 0.146	–	0.11
GA (wks), mean (range)	25.03 (23.1–26.6)	–	25.88 (23.6–26.6)	–	**0.003**
Apgar score ≤ 6 at 1 min	8/19	42.1	27/35	77.1	0.44
Discrete WMA at term age^a^	10/18	55.6	14/33	42.4	0.37
Patent ductus arteriosus, surgical ligation	12/19	63.2	5/35	14.3	** < 0.001**
Patent ductus arteriosus, Ibuprofen treated	13/19	68.4	22/35	62.9	0.68
Mechanical ventilation ≥ 10 days^b^	14/18	77.8	7/32	21.9	** < 0.001**
Mild–moderate lateral ventricular dilatation on cranial ultrasound^a^	5/18	27.8	7/33	21.2	0.60
Post-haemorrhagic ventricular dilatation	1/19	5.3	0/35	0.0	0.17
Antenatal steroids	17/19	89.5	34/35	97.1	0.24
Sepsis	17/19	89.5	21/35	60.0	**0.02**
SGA	1/19	5.3	3/35	8.6	0.66

Data in bold stand for a significance of *p*<0.05

*GA* gestational age, *wks* weeks, *BW* birthweight, *g* grams, *SD* standard deviation, *IVH* intraventricular haemorrhage, *WMA* white matter abnormalities, and *SGA* small for gestational age

^a^Available data: 18 children born extremely preterm with IVH, and 33 children born extremely preterm without IVH

^b^Available data: 18 children born extremely preterm with IVH, and 32 children born extremely preterm without IVH

During the infants' hospital stay, a qualified neonatologist routinely performed cranial ultrasounds and scored and evaluated IVH using Papile’s classification (Papile et al. 1978). Cranial ultrasound was used to detect IVH during the first 3 days following delivery until the 27th week of life, when it was subsequently done every other week until term-equivalent age was reached. Examinations were conducted more regularly if any pathology was identified. This study comprised low-grade IVH, specifically, grade I subependymal haemorrhage, and grade II intraventricular haemorrhage without dilatation. Brain MRI scans were performed at term-equivalent age and at 10 years old, and cognitive assessment was conducted at 12 years old. Yet, 9 children born extremely preterm and 11 children born at term declined or were unable to finish the cognitive assessment.

All participants’ parents provided written informed consent, and the regional ethics committee in Stockholm provided ethical approval.

### Cognitive assessment

The *Wechsler Intelligence Scale for Children V* was used to measure intellectual ability by assessing the following five indexes: verbal comprehension, visual spatial index, fluid reasoning, working memory, and processing speed. These indexes add up to a full-intelligence quotient (IQ) score (mean 100; SD 15) (Wechsler 2014).

According to Brøndbo and Egeland (2019), in a Scandinavian population, internal consistency for subtests and indexes was good to excellent. For the chosen five-factor model, goodness-of-fit analyses yielded a comparative fit index of 0.97–0.98 and a root-mean-square error of approximation of 0.05. The pattern of inter-correlations between subtests, process scores, and indexes was mainly similar to the standards used in the United States. The corrected rank correlation for full-IQ and the indexes WISC-V and WISC-IV were also high (0.67–0.91). Criteria validity, however, was lower than expected for predicting minor learning disabilities as well as skilled children.

All cognitive assessments were conducted by a certified psychologist (E.E.), who was blinded to the clinical history of each participant.

### MRI acquisition

The Philips Intera 1.5-T MRI system was used for imaging at term-equivalent age; the specifics of the sequence parameters have already been published (Skiöld et al. 2012). Another MRI scan of the study participants’ brain at 10 years of age was performed using a Sigma 3.0-T MR scanner (GE Healthcare) at Karolinska University Hospital, Sweden. The MRI protocol included a sagittal 3D-T1 weighted with a BRAVO SPGR sequence: time to inversion = 400 ms, field of vision = 240 × 240 mm^2^; flip angle = 12◦; voxel size 1 × 0.938 × 0.938 mm^3^; slice thickness = 1.0 mm.

### MRI images

Prior to processing, images were checked for movement and scanning artefacts. Hippocampal volumes were extracted from T1-weighted images using FreeSurfer (https://surfer.nmr.mgh.harvard.edu/) (version v6.0.0), and 3D-T1-weighted structural MRI scans were segmented to locate the hippocampus (Fischl 2012). T1 high-resolution images were processed using a variety of techniques, including intensity non-uniformity correction, skull stripping, affine transformation into MNI template, intensity normalisation, removal of non-brain tissue, linear and nonlinear transformations into a probabilistic brain atlas, and labelling of subcortical/allocortical structures. The appropriate label for each individual voxel was determined using spatial localisation priors (Fischl et al. 2002).

The FreeSurfer hippocampus subfield pipeline was used to analyse T1-weighted images through the hippocampal subfield segmentation programme FreeSurfer hippocampal-subfields-T1 command (Iglesias et al. 2015). The volumes of the right and left hippocampus were obtained. Each hippocampal hemisphere was also divided into 12 subfields, including the hippocampal tail, subiculum, presubiculum, parasubiculum, Cornu Ammonis 1 (CA1), CA2/3, CA4, granule cell layer of the dentate gyrus (GC-DG), molecular layer hippocampus (HP), fimbria, hippocampal–amygdalar transition area (HATA), and hippocampal fissure. Three grouped subfield volumes were created for each hippocampal hemisphere in accordance with previous researchers who have used this type of segmentation in preterm samples (Aanes et al. 2019; Fernández de Gamarra-Oca et al. 2021). This was also done, because our segmentations were based only on T1-weighted images and some hippocampal subfields are thought to be less reliable due to their size (Iglesias et al. 2015): CA-field (CA1 + CA2/3 + molecular layer HP + subiculum); Dentate gyrus (GC-DG + CA4); and Subiculum (presubiculum). Hippocampal volumetric values are expressed in mm^3^.

### Statistical analysis

Normal distribution of data was assessed using the Kolmogorov–Smirnov test (K–S). The Mann–Whitney *U* test was used to analyse differences in non-normally distributed data, such as GA, age at scan, and age at evaluation. The Chi-squared test was employed to assess differences in qualitative characteristics (i.e. clinical data, sex, handedness, and maternal education), and a Student's t test was run to compare birthweight (BW) between groups. To compare children born extremely preterm with their control peers in cognition (adjusted for maternal education) and hippocampal volumes (adjusted for ICV and sex), the Generalised Estimating Equations procedure was used as a generalised linear model. Bonferroni corrected p value for significance was calculated for both cognitive domains (*p* = 0.05/6 = 0.008) and global and hippocampal subfield volumes (*p* = 0.05/8 = 0.006).

Partial correlations (adjusted for sex and maternal education) were performed between the cognitive domains (i.e., verbal comprehension, visual spatial index, fluid reasoning, working memory, processing speed, and full-IQ) and global (right/left) as well as hippocampal subfield volumes, grouped into three subfield volumes for each hemisphere (i.e., CA-field, dentate gyrus, and subiculum) in both preterm groups independently and in the full-term group. To correct for multiple comparison purposes, Bonferroni corrected p value for significance was calculated (*p* = 0.05/14 = 0.004).

**Fig. 2:**
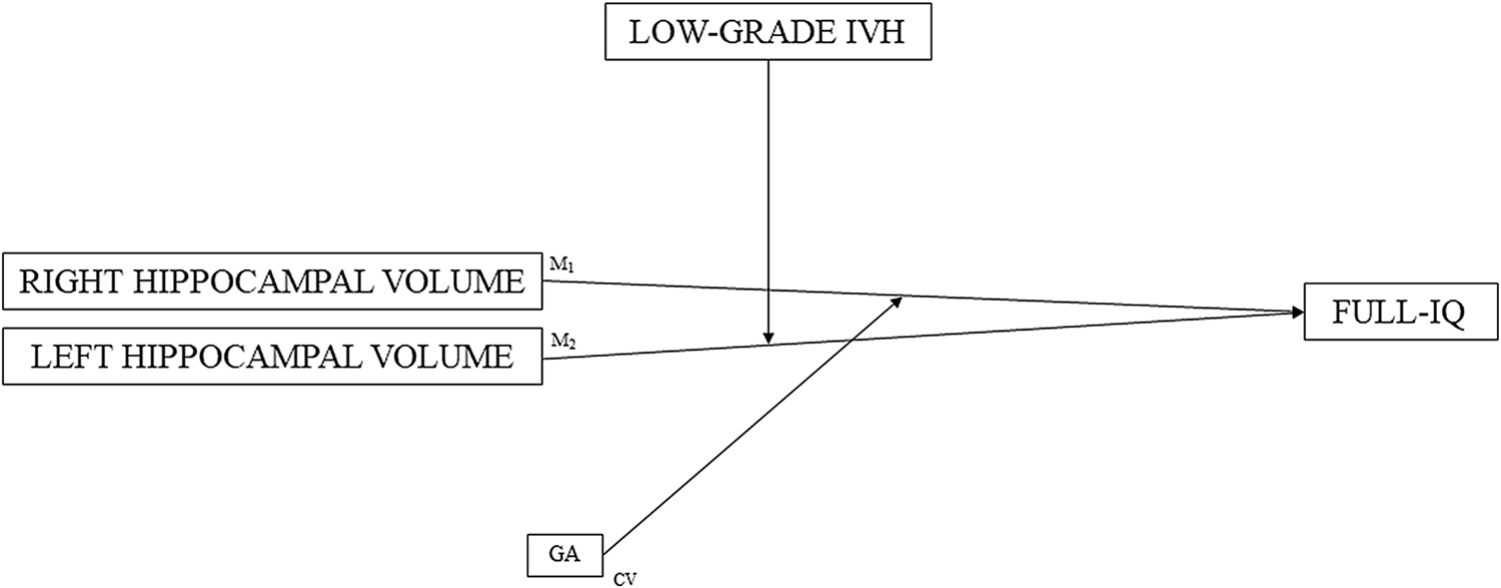
Moderation models between the hippocampal volumes and full-IQ in extremely preterm sample. GA: gestational age; IVH: intraventricular haemorrhage; and IQ: intelligence quotient. M_1_: the moderating effect of low-grade IVH in the relation between the right hippocampal volume and full-IQ adjusted for GA; and M_2_: the moderating effect of low-grade IVH in the relation between the left hippocampal volume and full-IQ adjusted for GA

Finally, the moderating effect of low-grade IVH was analysed in the relationship between right and left hippocampal volumes independently, and full-IQ in the extremely preterm sample (see Fig. 2). The macro-PROCESS 4.1 script for SPSS (which was made available on 19 April 2022) was used to run moderation analyses to measure the moderation effect. Given the differences in clinical data and the multicollinearity between these variables [i.e., GA, patent ductus arteriosus (surgical ligation), days on mechanical ventilation, and sepsis] in children born extremely preterm, the following analyses were adjusted only for GA. There were no obvious outliers in the study group, according to a scatterplot and histogram inspection. Moreover, before carrying out the moderation analyses, Mahalanobis and Cook’s distances as well as Leverage parameters were used to detect possible outliers. IBM SPSS version 28.0 (SPSS Inc., Chicago, USA) was used for all previous raw data analyses. Significance level was set to be below 0.05.

## Results

In the extremely preterm sample, there were significant differences in some clinical data between children born extremely preterm with and without low-grade IVH, respectively; specifically, in GA, patent ductus arteriosus (surgical ligation), days on mechanical ventilation, and sepsis. In contrast, no differences were found in sociodemographic variables (i.e. sex, age at scan, age at evaluation, handedness, and maternal education) between children born extremely preterm and the full-term group. Sociodemographic variables are detailed in Table 2. Differences were found in cognitive performance (see Table S1) between children born extremely preterm and their full-term peers, which remained significant after the Bonferroni correction was applied for multiple comparisons (*p* = 0.008).

**Table 2:** Sociodemographic data of children born extremely preterm and children born at term

	Children born extremely preterm *n* = 54 mean ± SD	Children born at term *n* = 40 mean ± SD	Statistics (*p*)
Male sex, *n*	26	19	*X*^2^ = 0.004 (0.95)
Age at scan. yrs [range]	10.28 ± 0.81 [9–11]	10.10 ± 0.77 [9–11]	*U* = 954.00 (0.34)
Age at evaluation^a^ yrs [range]	12.16 ± 0.23 [11–12]	12.12 ± 0.25 [11–12]	*U* = 629.50 (0.52)
Maternal education^b^ below university level/equal or more than university level	18/29	11/19	*X*^*2*^ = 0.02 (0.89)

Data in bold stand for a significance of *p*<0.05

*yrs* years, *U* Mann–Whitney *U* test, *X*^2^ Chi-square test, and *t* Student’s *t *test

^a^Available data: 46 children born extremely preterm, and 30 children born at term

^b^Available data: 47 children born extremely preterm, and 30 children born at term

### Aim 1: differences in hippocampal volumes at 10 years old

Significantly smaller volumes were found in the right and left subiculum of the hippocampus in children born extremely preterm compared to full-term controls. No significant differences between both groups were shown in other hippocampal subfields’ volumes (i.e., bilateral CA-field and bilateral dentate gyrus). In global volumetric analyses, no significant differences were found in right and left hippocampus between children born extremely preterm and the full-term group either (see Table 3).

**Table 3:** Global and hippocampal subfields’ differences between children born extremely preterm and children born at term

	Children born extremely preterm (*n* = 54) mean volume mm^3^ (SE)	Children born at term (*n* = 40) mean volume mm^3^ (SE)	Mean difference (confidence interval)	*P* value
*Hippocampal subfields (adjusted for ICV and sex)*				
Left CA-field	1830.28 (23.23)	1813.05 (20.71)	17.24 (− 45.01, 79.48)	0.59
Right CA-field	1861.98 (24.95)	1846.77 (19.08)	15.21 (− 49.26, 79.67)	0.64
Left dentate gyrus	540.77 (7.59)	543.03 (7.70)	− 2.26 (− 23.71, 19.19)	0.84
Right dentate gyrus	543.83 (6.82)	555.87 (7.69)	− 12.05 (− 32.47, 8.38)	0.25
Left subiculum	312.91 (4.63)	335.61 (4.34)	− 22.70 (− 34.60, − 10.80)	** < 0.001**
Right subiculum	302.50 (4.20)	323.66 (4.53)	− 21.16 (− 33.39, − 8.93)	**0.001**
*Global hippocampus (adjusted for ICV and sex)*				
Left hippocampus	3433.08 (38.30)	3430.25 (33.89)	2.83 (− 98.67, 104.33)	0.96
Right hippocampus	3444.60 (39.49)	3467.53 (31.95)	− 22.94 (− 125.55, 79.68)	0.66

*SE* standard error, and *ICV* intracranial volume

The hippocampal subfields in bold are those that remained significant after Bonferroni correction was applied for multiple comparisons (*p* = 0.006)

### Aim 2: correlation analyses between hippocampal volumes and cognition

**Fig. 3:**
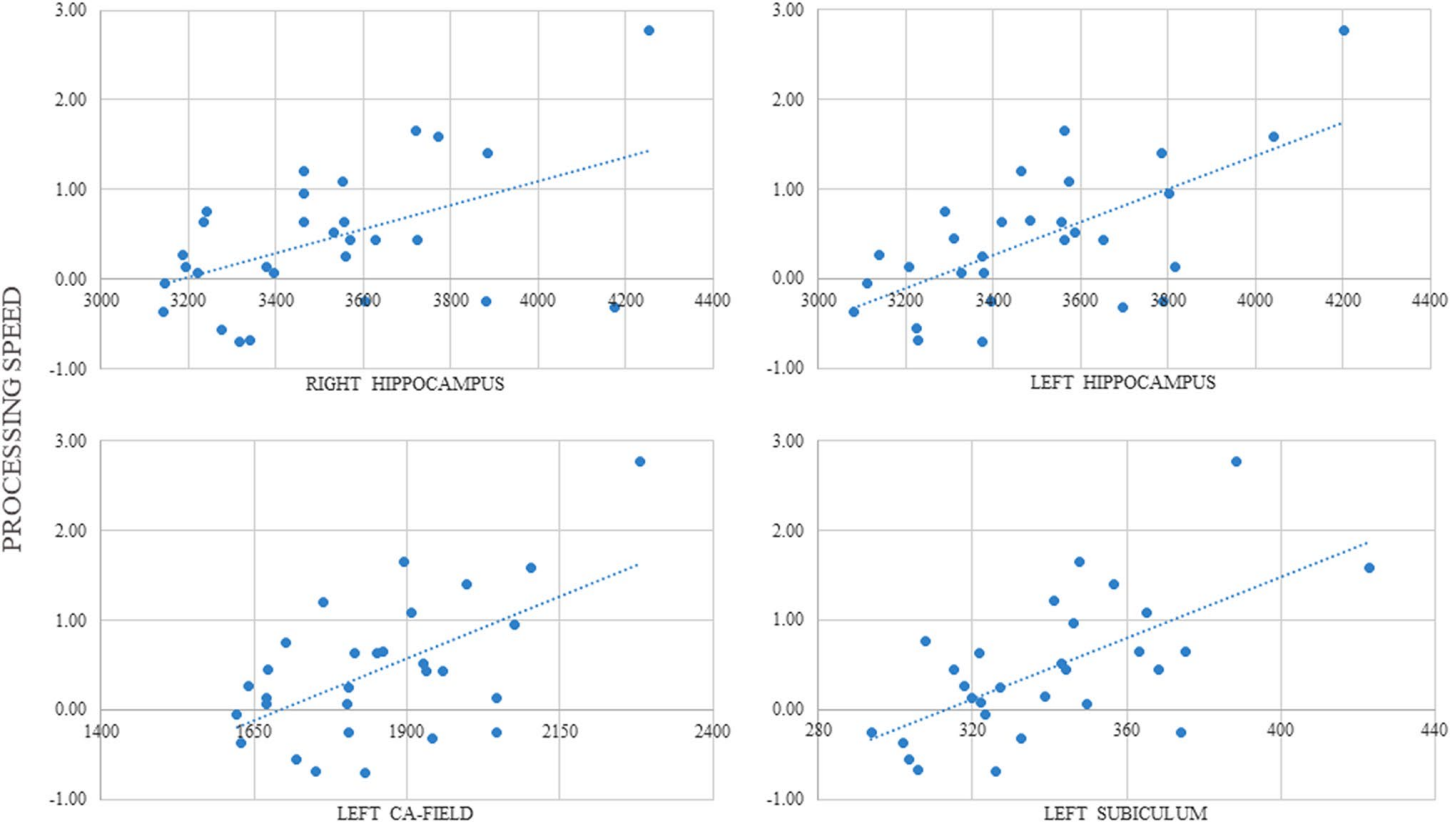
Partial correlations between hippocampal volumes and processing speed in children born at term. Details of partial correlations in children born at term significant correlations were observed between right hippocampus, left hippocampus, left CA-field, and left subiculum volumes with processing speed after Bonferroni correction for multiple comparisons was applied (*p* = 0.004). Unstandardized residuals were calculated and used for display purposes (adjusted for sex and maternal education)

Given that we further assessed cognitive performance when they were 12 years old, we examined correlations between hippocampal volumes (at age 10) and later cognition. When analysing extremely preterm children with and without low-grade IVH independently (see Tables S2 and S3), correlations were found between global and hippocampal subfield volumetric measures and cognition, but none remained significant after applying a Bonferroni correction for multiple comparisons. In children born at term, significant correlations were seen between global right (*r* = 0.567 *p* = 0.002) and left (*r* = 0.665 *p* < 0.001) hippocampus, and left CA-field (*r* = 0.586 *p* = 0.001) and left subiculum (*r* = 0.636 *p* < 0.001) volumes with processing speed remaining significant following the Bonferroni correction (see Fig. 3 and Table S4).

### Aim 3: moderation effect of low-grade IVH

**Fig. 4:**
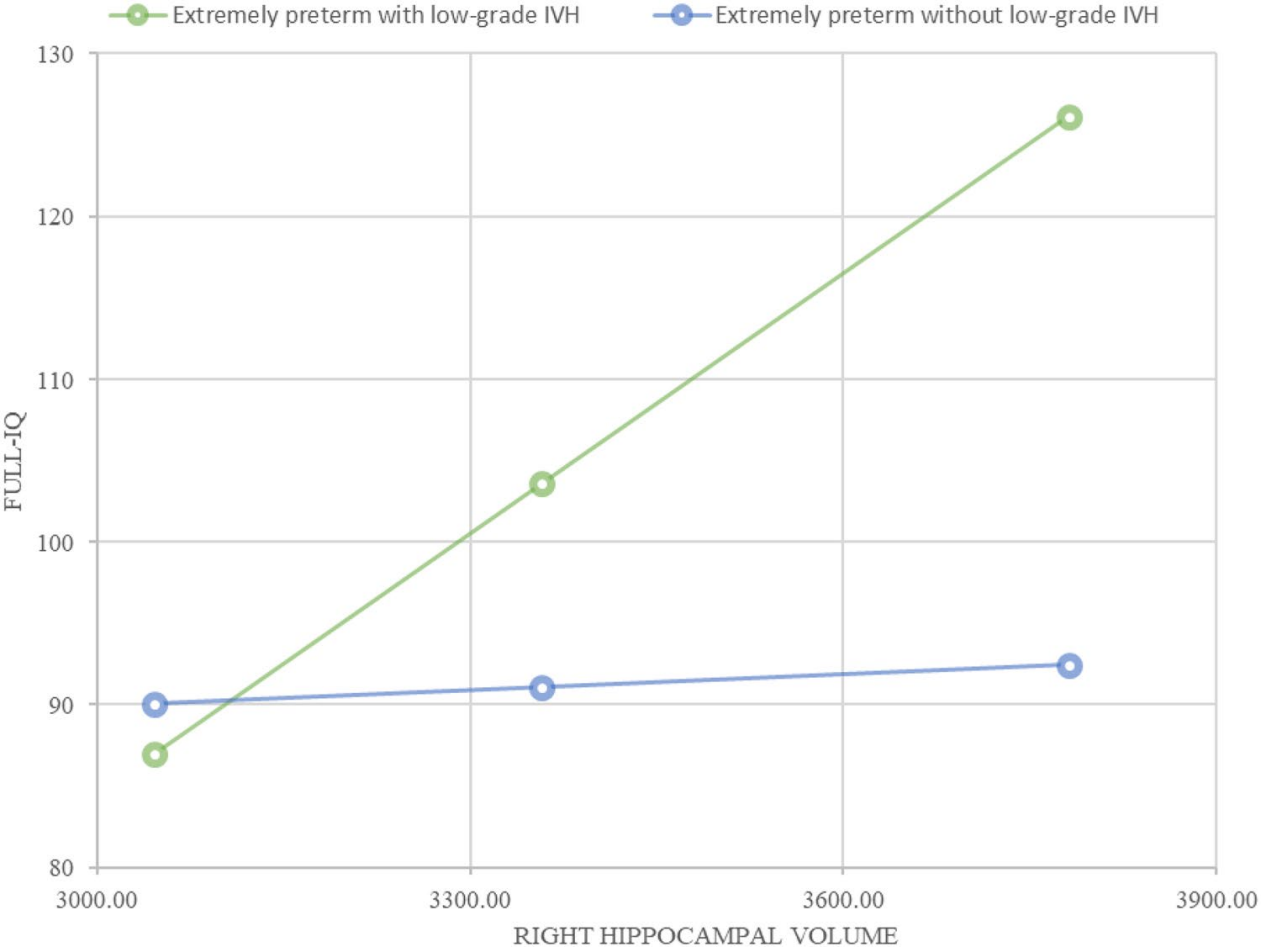
Moderating effect of intraventricular haemorrhage I-II between right hippocampal volume and full-IQ in extremely preterm sample. Low-grade IVH showed a significant relationship between the right hippocampal volume and full-IQ across childhood (green line); whereas not suffering from low-grade IVH did not moderate a relationship between the right hippocampal volume and full-IQ (blue line). The presence of low-grade IVH leads to a significant relationship between small, medium or great right hippocampal volume and full-IQ across childhood; leading to an enhanced full-IQ as right hippocampal volume increases in extremely preterm children with low-grade IVH (adjusted for GA)

Finally, in moderation analyses (see Fig. 4), we tested the pre-specified hypothesis that the presence/absence of low-grade IVH would have a moderating effect on full-IQ for the entire extremely preterm group based on their hippocampal volumes. We found that 35% of the variance was explained by the three factors (i.e., presence/absence of low-grade IVH, right hippocampal volume, and the interaction of both) in the overall model (*F (*_4,40) =_5.42, *p* = 0.001). More specifically, the presence of low-grade IVH moderated the structural–functional relationship between great right hippocampal volume (i.e., 3781.43 mm^3^) and full-IQ. Nevertheless, having either a medium or small right hippocampal volume was also significantly related to full-IQ in the extremely preterm group with low-grade IVH. In contrast, while the overall moderation analysis model of left hippocampal volume in relation to full-IQ was significant, the non-significant interaction between predictors showed the lack of moderation effect.

## Discussion

By considering well-characterised groups of children born extremely preterm with and without low-grade IVH, this study emphasises the existence of different brain mechanisms depending on right hippocampal volume during childhood. Moreover, reduced bilateral subiculum subfield volumes were found only in those born extremely preterm, which confirms a biological predisposition in early life that causes the hippocampus to be more vulnerable because of unfavourable perinatal conditions (Strahle et al. 2019; Thompson et al. 2008). However, contrary to expectations, memory performance (i.e., working memory) was not explained by regional hippocampal volumetric differences, since this is not the only structure involved. For instance, in typically developing neonates, hippocampal functional connectivity with adjacent limbic and subcortical regions, as well as increasing connectivity with default mode network regions by the end of the first year, predicted working memory performance at 4 years old (Liu et al. 2021).

Our findings showed lower scores in cognition in children born extremely preterm. Given that up to 73% of the children who were born extremely preterm had mild or no disability at 2.5 years old, it made sense that their cognitive performance was within the normal range (Serenius et al. 2013). However, early developmental deficits in children born extremely preterm do not seem to improve overtime (Pascoe et al. 2021).

The hippocampus and its related regions may suffer from long-lasting structural abnormalities after very preterm birth (Nosarti et al. 2016). Nevertheless, our results did not show reduced global hippocampal volumes in children born extremely preterm. Even though the subiculum is particularly vulnerable to hypoxic–ischemic episodes (Stark 2007), this study found hippocampal subfield volumetric reductions in bilateral subiculum in children born extremely preterm with and without low-grade IVH. Nonetheless, the typical growth experienced by this structure might also be negatively influenced by clinical conditions seen in preterm newborns, such as the number of days on mechanical ventilation in preterm adults with very low BW (Aanes et al. 2020). However, our study sample only included four children with SGA. According to O’Mara et al. (2009), the subiculum codes space in a qualitatively different manner than the hippocampus, complementing hippocampal-based spatial information processing but not relying only on hippocampal input. Therefore, the subiculum has been seen as a hippocampus hub with a special role in information processing (Matsumoto et al. 2019).

It is well known that those who are born extremely preterm are more likely to have poor cognitive performance, which has been linked to altered brain development (Baron et al. 2010). While we observed volume reductions of bilateral subiculum subfield volumes after extremely preterm birth during childhood, functional associations were more complex. Whereas the current study found robust relationships between global and regional hippocampal volumes and processing speed among children born at term, this was not the case for working memory. It has been suggested that the hippocampus plays a role not only in episodic memory, working memory, and the executive function, but also in processing speed across aging (O’Shea et al. 2016). Unlike many previous studies (Aanes et al. 2019, 2020; Fernández de Gamarra-Oca et al. 2021), although subiculum subfield growth was affected due to extremely preterm birth, our study did not show cognition correlates. Further, low-grade IVH moderated the relationship between right hippocampal volume at 10 years old and full-IQ during late childhood. That is, great right hippocampal volume has a protective effect on full-IQ of children born extremely preterm with low-grade IVH. As per some authors, even with low-grade IVH, infants born preterm may experience a negative impact on their neurodevelopment (Bolisetty et al. 2014; Hollebrandse et al. 2021; Klebermass-Schrehof et al. 2012; Patra et al. 2006).

## Limitations

The relatively small sample size, given the characteristics of the children, as well as the fact that not all of those with MRIs had undergone cognitive assessment, were some of the study's limitations. More research is therefore needed to prove the relationship of hippocampal volumetric values with cognition in larger and longitudinally followed up populations of children born extremely preterm, with and without low-grade IVH. Nevertheless, our study followed clear standards to ensure adequate comparison groups while considering maternal education and clinical data. Furthermore, because only low-grade IVH was included, it is impossible to determine if hippocampus volume and cognitive performance may be affected by the different grades suggested by Papile et al. (1978). It is clear that the risk for prenatal morbidities and, notably, negative long-term cognitive outcomes, increase with grade severity (Inder et al. 2018). However, as there is no extensive literature focusing on low-grade IVH, this may also be seen as a strength. Despite these weaknesses, this study identifies a moderation effect of low-grade IVH in a structural–functional relationship in a well-characterised population born extremely preterm.

## Conclusions

Poorer cognitive performance and reduced subiculum subfield volumes were observed in a sample of school-aged children born extremely preterm, with and without low-grade IVH. Knowing the process through which prematurity-related adverse neurocognitive outcomes develop increases the likelihood of early detection of children thought to be at a higher risk. Moreover, a strong association between global and regional hippocampus volumes and processing speed was found in children born at term, even though hippocampus–cognition relationships were not observed in children born extremely preterm. However, as the neuroanatomical basis for cognition is undoubtedly complex, hippocampal volumetric variations alone might not fully explain functional performance. Finally, only children born extremely preterm with low-grade IVH were influenced by right hippocampal volumes in relation to their full-IQ during late childhood. Nonetheless, more studies pondering low-grade IVH are required to learn about its possible impact and the potential compensatory processes linked to cognition.

## Supplementary Information

Below is the link to the electronic supplementary material.

Supplementary file1 (DOCX 60 KB)

## Data Availability

The data are available from the corresponding author upon reasonable request.
