# Supplementary material for: Hippocampal volumes and cognitive performance in children born extremely preterm with and without low-grade intraventricular haemorrhage
Source: Brain Struct Funct. 2023 Apr 21;228(5):1191–200. doi: 10.1007/s00429-023-02643-w (PMC10250428; doi:10.1007/s00429-023-02643-w)
Supplement: Supplementary file 1 — Supplementary file1 (DOCX 60 KB) [file 429_2023_2643_MOESM1_ESM.docx]

**Supplementary material**

Table S1

*WISC V Differences between Children Born Extremely Preterm and Full-Term Controls*

|  | Children born extremely preterm (n=45)  mean score (SE) | Children born at term  (n=29)  mean score (SE) | Mean difference (confidence interval) | P-value |
| --- | --- | --- | --- | --- |
| ***WISC-V Scales’ Assessment*** (adjusted for maternal education) | | |  |  |
| Verbal Comprehension | 100.50 (2.58) | 116.69 (2.80) | -16.19 (-23.66, -8.71) | **<0.001** |
| Visual Spatial Index | 93.38 (2.36) | 104.45 (2.35) | -11.07 (-17.44, -4.69) | **0.001** |
| Fluid Reasoning | 95.59 (1.81) | 105.48 (1.98) | -9.89 (-15.05, -4.73) | **<0.001** |
| Working Memory | 90.17 (2.14) | 99.25 (1.94) | -9.08 (-14.77, -3.39) | **0.002** |
| Processing Speed | 93.76 (2.45) | 106.18 (2.70) | -12.43 (-19.54, -5.32) | **0.001** |
| Full-IQ | 95.22 (2.23) | 110.55 (2.21) | -15.33 (-21.49, -9.17) | **<0.001** |

**Note**: SE: standard error; WISC-V: Wechsler Intelligence Scale for Children V; and IQ: intelligence quotient.

The cognitive domains in bold are those that remained significant after Bonferroni correction was applied for multiple comparisons (*p*=0.008).

Table S2

*Partial Correlations between Hippocampal Volumes and Cognitive Domains in Children Born Extremely Preterm with Intraventricular Haemorrhage I-II*

| Criterion variable | Verbal comprehension | Visual spatial index | Fluid reasoning | Working memory | Processing speed | Full-IQ |
| --- | --- | --- | --- | --- | --- | --- |
| Left CA-field | .227 | .311 | .136 | .307 | .123 | .329 |
| Right CA-field | .192 | .380 | .535* | .259 | .553* | .474 |
| Left Dentate Gyrus | .059 | .119 | -.140 | .226 | -.062 | .055 |
| Right Dentate Gyrus | .146 | .390 | .436 | .166 | .399 | .371 |
| Left Subiculum | .485 | .527* | .601* | .342 | .323 | .562* |
| Right Subiculum | .425 | .562* | .607* | .506* | .552* | .650** |
| Left Hippocampus | .351 | .372 | .204 | .257 | .188 | .393 |
| Right Hippocampus | .351 | .457 | .531* | .205 | .493 | .517* |

Significance level **p*<.05; ***p*<0.01.

In bold those that remained significant after Bonferroni correction was applied for multiple comparisons (*p*=0.004).

Table S3

| Criterion variable | Verbal comprehension | Visual spatial index | Fluid reasoning | Working memory | Processing speed | Full-IQ |
| --- | --- | --- | --- | --- | --- | --- |
| Left CA-field | .112 | .140 | .205 | .165 | .212 | .256 |
| Right CA-field | .160 | .254 | .249 | .252 | .248 | .325 |
| Left Dentate Gyrus | .137 | .178 | .256 | .220 | .194 | .289 |
| Right Dentate Gyrus | .181 | .226 | .316 | .283 | .275 | .400* |
| Left Subiculum | .044 | -.117 | -.221 | -.152 | .054 | -.076 |
| Right Subiculum | .163 | .059 | -.029 | .111 | .228 | .191 |
| Left Hippocampus | .118 | .179 | .200 | .175 | .225 | .226 |
| Right Hippocampus | .155 | .246 | .242 | .248 | .236 | .307 |

*Partial Correlations between Hippocampal Volumes and Cognitive Domains in Children Born Extremely Preterm without Intraventricular Haemorrhage I-II*

Significance level **p*<.05; ***p*<0.01.

In bold those that remained significant after Bonferroni correction was applied for multiple comparisons (*p*=0.004).

Table S4

*Partial Correlations between Hippocampal Volumes and Cognitive Domains in Children Born at Term*

| Criterion variable | Verbal comprehension | Visual spatial index | Fluid reasoning | Working memory | Processing speed | Full-IQ |
| --- | --- | --- | --- | --- | --- | --- |
| Left CA-field | -.254 | .169 | -.065 | .083 | **.586** | .009 |
| Right CA-field | -.266 | .189 | .023 | .191 | .522** | .054 |
| Left Dentate Gyrus | -.194 | .283 | -.008 | .028 | .505** | .081 |
| Right Dentate Gyrus | -.263 | .152 | .189 | .202 | .491** | .114 |
| Left Subiculum | -.081 | .044 | .041 | .306 | **.636** | .133 |
| Right Subiculum | -.135 | -.067 | .045 | .356 | .413* | .079 |
| Left Hippocampus | -.253 | .188 | -.034 | .118 | **.665** | .041 |
| Right Hippocampus | -.283 | .166 | .013 | .245 | **.567** | .044 |

Significance level **p*<.05; ***p*<0.01.

In bold those that remained significant after Bonferroni correction was applied for multiple comparisons (*p*=0.004).
